# Reproducible machine learning research in mental workload classification using EEG

**DOI:** 10.3389/fnrgo.2024.1346794

**Published:** 2024-04-10

**Authors:** Güliz Demirezen, Tuğba Taşkaya Temizel, Anne-Marie Brouwer

**Affiliations:** ^1^Department of Information Systems, Graduate School of Informatics, Middle East Technical University, Ankara, Türkiye; ^2^Department of Data Informatics, Graduate School of Informatics, Middle East Technical University, Ankara, Türkiye; ^3^Human Performance, Netherlands Organisation for Applied Scientific Research (TNO), Soesterberg, Netherlands; ^4^Donders Institute for Brain, Cognition and Behaviour, Radboud University, Nijmegen, Netherlands

**Keywords:** neuroergonomics, reproducibility, EEG, physiological measurement, mental workload, machine learning, brain-computer interface, neuroscience

## Abstract

This study addresses concerns about reproducibility in scientific research, focusing on the use of electroencephalography (EEG) and machine learning to estimate mental workload. We established guidelines for reproducible machine learning research using EEG and used these to assess the current state of reproducibility in mental workload modeling. We first started by summarizing the current state of reproducibility efforts in machine learning and in EEG. Next, we performed a systematic literature review on Scopus, Web of Science, ACM Digital Library, and Pubmed databases to find studies about reproducibility in mental workload prediction using EEG. All of this previous work was used to formulate guidelines, which we structured along the widely recognized Cross-Industry Standard Process for Data Mining (CRISP-DM) framework. By using these guidelines, researchers can ensure transparency and comprehensiveness of their methodologies, therewith enhancing collaboration and knowledge-sharing within the scientific community, and enhancing the reliability, usability and significance of EEG and machine learning techniques in general. A second systematic literature review extracted machine learning studies that used EEG to estimate mental workload. We evaluated the reproducibility status of these studies using our guidelines. We highlight areas studied and overlooked and identify current challenges for reproducibility. Our main findings include limitations on reporting performance on unseen test data, open sharing of data and code, and reporting of resources essential for training and inference processes.

## 1 Introduction

Reproducibility is fundamental for research advancement. Reproducing results, not only by the owners of the original study but also by other researchers, enables establishing a solid foundation that can be built upon for global research progress. The ability to repeat a study of others using the exact same methodology and produce the same results facilitates the verification and validation of study findings, identification or reduction of errors, and accurate comparison of newly developed methodologies. This process not only increases the trustworthiness of findings but also bolsters the credibility of the researchers involved and science in general. Moreover, reproducibility ensures the seamless deployment and long-term usability of applications.

However, findings suggest that most research is not reproducible. A Nature survey, for instance, revealed that 70% of researchers could not reproduce another researcher's experiments, while over 50% could not reproduce their own research (Baker, 2016). Gundersen and Kjensmo (2018) investigated the reproducibility status of 400 papers from the IJCAI and AAAI conference series and concluded that only approximately 25% of the variables required for reproducibility were adequately documented. A systematic and transparent reporting is essential to support reproducibility.

In the rapidly evolving field of neuroergonomics and Brain-Computer Interface (BCI) applications, the need to ensure the reproducibility of research findings is high. Most neuroergonomic and BCI applications require artificial intelligence (AI) and machine learning (ML) technologies to identify patterns in brain signals with the aim of decoding user's intentions or distinguishing mental states. For example, these techniques are used in passive BCIs to evaluate mental workload in real time and adapt the tasks using the estimated workload, which could be beneficial for monitoring and supporting professionals whose work requires high focus. It can also aid in selecting alternatives for human-computer interaction (HCI) systems that induce the least amount of load on the users. Although the evaluation of mental workload from EEG signals is extensively studied in the literature (Saeidi et al., 2021), many challenges remain to be addressed toward developing real-life applications. These challenges include ability to generalize across subjects (Roy et al., 2013), across sessions or over time (Millan, 2004; Roy et al., 2022), across tasks and across contexts (Mühl et al., 2014; Lotte et al., 2018; Hinss et al., 2023). A systematic and reproducible approach can foster a more collaborative research environment, enabling more effective and rapid solutions to these challenges.

Despite increased attention for reproducibility in the literature, definitions of reproducibility remain unclear and even conflicting (National Academies of Sciences Engineering and Medicine, 2019). In this paper, we adopt the definition from (p. 1645) Gundersen and Kjensmo (2018) as expressed for reproducibility in AI, which was set forth as “*Reproducibility in empirical AI research is the ability of an independent research team to produce the same results using the same AI method based on the documentation made by the original research team.”*. Achieving reproducibility necessitates documenting research at a certain level of detail. Gundersen and Kjensmo (2018) grouped documentation into method, data, and experiment categories. They also defined three levels of reproducibility, namely R1: Experiment Reproducible, R2: Data Reproducible, and R3: Method Reproducible. R1 reproducibility necessitates sharing all three documentation categories. In R1, the results are expected to be the same, except for minor differences due to hardware changes, as the same implementation is executed on the same data. R1 corresponds to fully reproducible research (Peng, 2011) and technical reproducibility (McDermott et al., 2021). As the reproducibility level increases from R1 to R2 and R3, the generalizability of the models increases, and documentation requirements decrease at the cost of reduced transparency. The generalizability of a model is the degree to which the outcomes of a study are applicable to diverse contexts or populations. R2 reproducibility is attributed when an alternative implementation of the method is executed on the same data, hence requiring the openness of method description and data but not the scripts, and it is generalizable to alternative implementations of the method. Finally, R3 reproducibility is expected to yield the same results with alternative implementations on different data, thus necessitating only the method documentation. Obtaining similar performance in this case is a step in concluding that the improvement of research was made possible by the proposed method, and the method is generalizable. It should be noted that reproducibility does not necessarily guarantee accuracy. Even if the results are not favorable, the study can be reproducible. Moreover, there is not a single best solution for a given problem, which is another reason for detailed reporting (Pernet et al., 2020).

In this study, we propose guidelines considering full (R1) reproducibility with the aim of maximum transparency, enabling the generation of the same results with the same implementation and on the same data. This level of sharing can be tailored for R2 reproducibility level by leaving out the reporting of the experiment and for R3 reproducibility level by leaving out the reporting of both the experiment and data. These approaches reduce the degree of reproducibility but are steps toward generalizable solutions. In cases where the scripts or data are not made available, authors need to be willing to assist other researchers in constructing the baseline (Collberg and Proebsting, 2016).

While guidelines for the reproducibility of machine learning and EEG studies exist independently in the literature, there is a lack of integrated guidance covering both. EEG guidelines primarily emphasize standardized procedures for data collection, preprocessing, sharing, and statistical analysis. Recommendations for machine learning stress best practices in feature engineering, modeling, and evaluation and highlight code transparency and dataset availability. The necessity to connect these guidelines becomes apparent with the rising number of publications employing machine learning on EEG data, combined with the already mentioned challenges in the generalizability of EEG-based mental state estimations across subjects and contexts or over time. In the current manuscript, we aim to close this gap in the literature and combine a reproducible and standardized ML pipeline with EEG guidelines with a focus on the classification of mental workload. Based on previous work, we establish guidelines and a checklist for reproducible EEG machine learning. Using this checklist, we systematically assess to what extent studies currently adhere to this checklist.

To scope our research, we chose to focus on workload recognition since it represents a substantial and relatively well-defined sub-area of mental state monitoring and passive BCI. In fact, workload and multitasking emerged as the most common mental state or process, according to the survey conducted by Putze et al. (2022).

The proposed guidelines and checklist have the potential to be applicable to most other types of EEG ML mental state assessment studies. However, the specific nuances of each domain must be considered during implementation.

Our manuscript is outlined as follows. In Section 2, we first introduce the current reproducibility status in machine learning and explain the CRISP-DM methodology, which is a commonly used standard for data mining machine learning projects. Then, we present the reproducibility efforts in EEG studies (Section 3) and systematically review papers that studied reproducibility in mental workload prediction using EEG (Section 4). In Section 5, we combine the findings in the literature with our contributions and propose guidelines for a reproducible EEG machine learning pipeline that is incorporated into the CRISP-DM phases. Following from these guidelines, we then create a compiled checklist of the requirements for reproducibility. In Section 6, adhering to the proposed checklist, we assess the current reproducibility status of machine learning models that utilize EEG to measure mental workload based on a comprehensive systematic literature review. We performed both systematic literature reviews following the Preferred Reporting Items for Systematic Reviews and Meta-Analyses (PRISMA) flow diagram (Moher et al., 2010). Finally, Section 7 is allocated for the discussion.

## 2 Reproducibility in machine learning

In research fields where machine learning solutions are applied, the challenge of reproducibility is prominent. Independent researchers often struggle to replicate the same results solely based on information provided in publications (Baker, 2016; Gundersen and Kjensmo, 2018; Hutson, 2018). In light of recent discussions on the reproducibility crisis, efforts to examine reproducibility in publications and introduce guidelines or checklists have expanded. Organizations and academic publishers have developed reproducibility checklists to ensure that research incorporates a minimum set of essential information and statistical checks, promoting openness in order to transparently report reproducible work (Kenall et al., 2015). Leading journal editors, funding agencies, and scientific leaders collaboratively established a comprehensive set of Principles and Guidelines in Reporting Preclinical Research in June 2014 (McNutt, 2014), and a considerable number of journals have agreed to support it. These principles include rigorous statistical analysis and transparency in reporting together with a proposed set of key information and data and material sharing. Academic organizations have also introduced checklists to promote reproducibility in machine learning studies. For example, Pineau et al. (2021) generated “The Machine Learning Reproducibility Checklist” which was used in NeurIPS 2019. This checklist includes items for models and algorithms, theoretical claims, and figures and tables. Authors emphasize significant cultural and organizational changes besides code submission policy or a checklist to achieve reproducibility. As discussed in the previous section, Gundersen and Kjensmo (2018) curated a checklist to investigate the status of reproducibility. Following specific guidelines facilitates a systematic process for conducting reproducible research.

The most widely used methodology for structuring data mining machine learning projects is the CRISP-DM. Introduced in 2000, CRISP-DM is a baseline process model to define and standardize data science life cycle in industry (Chapman et al., 2000). This iterative process comprises six phases, each of which is briefly explained below. We use these phases to structure our checklist in Section 5.

1. Business Understanding: The initial phase aims to identify business objectives, metrics, and success criteria for subsequent model evaluation. Additionally, it involves defining and planning available resources, as well as establishing strategies to mitigate potential project risks throughout the project lifecycle. In addition to these fundamental project management activities, data mining objectives and corresponding technical success criteria are determined during the business understanding phase. Finally, a project plan is devised for each subsequent phase of the project, ensuring a cohesive and strategic approach.
2. Data Understanding: This phase consists of the tasks of data collection, data description, data exploration, and data quality verification. Data collection adheres to established best practices within the relevant domain, with a clear presentation of data definitions, types, and additional requirements. This phase also entails the examination of data for cleanliness, addressing issues like missing values, noise, outliers, and data imbalance. In this phase, data is understood, and subject matter knowledge is acquired so that each member of the project has a common ground on terminology and domain knowledge. Moreover, future decisions on data preparation, modeling, evaluation and deployment can be made informed only if the context specific to the domain is well understood.
3. Data preparation: This phase involves organizing data for modeling purposes. Tasks encompass data selection based on goals and limitations, which may include technical or quality considerations. Additionally, this phase includes data cleaning, filtering, and the creation of new attributes or samples through data transformation, augmentation, and integration from multiple sources. Feature engineering and selection are also integral components of this phase.
4. Modeling: The modeling phase starts with the selection of an appropriate method tailored to the specific problem. The rationale behind this selection and any underlying modeling assumptions need to be documented. Reasons to select an algorithm may be related to data and problem characteristics or may arise from some constraints such as development time or hardware limitations. Certain methods incorporate feature selection, which can be another factor to take into account. After the selection of the modeling technique, test design is performed, and model building is initiated. As models are developed, they are assessed and ranked based on predefined evaluation criteria, also taking into account the business success criteria when possible. Model parameters are adjusted iteratively based on these evaluations until a satisfactory model is achieved.
5. Evaluation: The model's compliance to predefined business objectives is assessed in this phase rather than the model performance that was considered in the previous modeling phase. Testing the models in deployment environments can also be anticipated. If the results prove to be insufficient, it may be necessary to revisit earlier phases. This could entail fine-tuning the hyperparameters, exploring alternative algorithms, or reevaluating data preparation and conducting more comprehensive data exploration. Upon achieving satisfactory results in the evaluation phase, a final review of the process is necessary to address any potential oversights. Favorable outcomes from this review pave the way for the subsequent deployment stage.
6. Deployment: At the beginning of this phase, a strategy for deployment is developed. This phase highlights the significance of generalization, as the system or solution is implemented in real-world settings. Here, inference is done on novel data which was never encountered by the model previously. The model's ability to adapt to various scenarios and different users is put to test. Constructing and thoroughly testing the deployment environment is a crucial component of this phase. Furthermore, this phase involves meticulous planning for the continuous monitoring and maintenance of the system. Considering the evolving nature of businesses, model drift may occur, necessitating the retraining of the model with recent data to capture updated business aspects.

Various other data process models besides CRISP-DM are available, such as “Knowledge Discovery in Databases (KDD)” (Fayyad et al., 1996), “Sample, Explore, Modify, Model, Assess (SEMMA)^1^” and “Team Data Science Process (TDSP)^2^” are available. We chose to use CRISP-DM not only because it is widely adopted (Schröer et al., 2021), but also because its phases align well with EEG processing and the machine learning pipeline and because it covers “Business Understanding” and “Deployment” phases, which are necessary to build applications. “Business Understanding” and “Deployment” phases are not included in KDD or SEMMA models. While phases of CRISP-DM and TDSP are similar, CRISP-DM incorporates more detailed phases related to data processing, modeling and evaluation which are fundamental steps for conducting machine learning studies using EEG.

## 3 Reproducibility in EEG

Reproduction of EEG studies comes with challenges, some of which are inherent to scientific research, while others arise from the nature of EEG data. Variations in data collection settings, such as the environment, electrode placement, or online filters, can lead to differences in results. Individuals differ in terms of anatomical and neurophysiological characteristics. Order of preprocessing steps and a large number of parameters that are used within different preprocessing methodologies can cause large differences (Robbins et al., 2020). These problems can be mitigated through systematic and transparent reporting. In EEG research, there is a considerable number of publications that aim to standardize data formats, data collection methodologies, data analysis (particularly statistical analysis and preprocessing), and data sharing.

The Brain Imaging Data Structure (BIDS) standard was developed to standardize MRI datasets by defining file structure, format, and naming conventions as well as guidelines for presenting metadata (Gorgolewski et al., 2016). Pernet et al. (2019) established EEG-BIDS to introduce this standard to the EEG domain. Specific to EEG data, they recommended the European Data Format (EDF) and the BrainVision Core Data Format, alongside allowing two unofficial data formats due to their common usage and to ease adoption of EEG-BIDS: EEGLAB's format (“.set” and “.fdt” files) and the Biosemi format (“.bdf”).

In 2014, a committee appointed by the Society for Psychophysiological Research reported comprehensive guidelines for studies using EEG and MEG with a detailed checklist for reporting (Keil et al., 2014). The covered topics are hypotheses, participants, recording characteristics and instruments, stimulus and timing parameters, data preprocessing, measurement procedures, figures, statistical analysis, spectral analysis, source-estimation procedures, Principle Component Analysis (PCA) and Independent Component Analysis (ICA), multimodal imaging, current source density and Laplacian transformations, and single-trial analyses. The Organization for Human Brain Mapping (OHBM) neuroimaging community (Committee on Best Practices in Data Analysis and Sharing (COBIDAS) MEEG - where MEEG refers to MEG and EEG) compiled best practices for data gathering, analysis, and sharing (Pernet et al., 2020). Recommendations encompassed MEEG data acquisition and data analysis terminologies, definitions, and basic experimental attributes to include in an article. They also listed MEEG preprocessing and MEEG connectivity modeling parameters to be reported and their impact on reproducibility. The authors also state the importance of a dynamic guideline, which is to be adapted as new technology and methods arise. Similarly, Kane et al. (2017) included the most commonly used clinical EEG terms and proposed a standardized and structured EEG report form.

Putze et al. (2022) created an overarching experiment model that provides a formal structure for presenting HCI research using brain signal data to enhance reproducibility and reusability. They further conducted statistical analysis to understand reporting structures and identified reporting gaps for 110 papers from dedicated HCI conferences or journals. The recommendations and discussions on future challenges offer valuable insights for the advancement of HCI practices. While the focus of this publication was on HCI, the aspects they list are mostly applicable to EEG ML studies in general.

We refer to these established EEG guidelines to develop a compiled checklist in Section 5.

## 4 Reproducibility in mental workload studies using EEG

In this section, we present the outcomes of our systematic literature review on reproducibility studies related to EEG and mental workload. Our aim is to determine to what extent studies that use EEG to measure mental workload have focused reproducibility.

### 4.1 Literature search strategy

**Figure 1:**
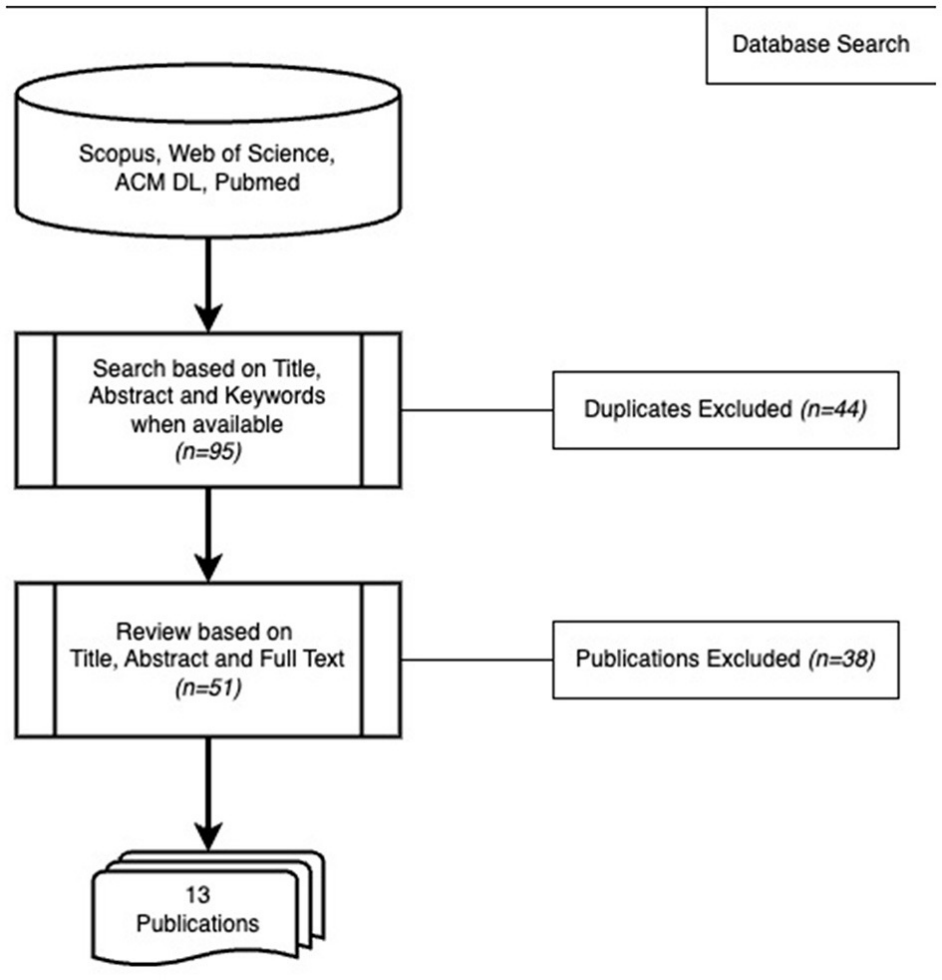
Search strategy for literature review 1.

Figure 1 shows the search strategy. In phase I, we conducted a search using specific terms across Scopus, Web of Science, ACM Digital Library (ACM DL), and Pubmed databases to assess the current state of reproducibility in this field. We searched in titles, abstracts, and keywords with the following search term: *(“Reproducibility” OR “Replicability” OR “Generalizability”) AND “EEG” AND (“Workload” OR “Cognitive Load” OR “Mental Effort” OR “Mental Load”)* in February 2024. The search was constrained to a publication year up to and including 2023 at the latest.

### 4.2 Eligibility criteria

In phase II, we considered a publication relevant according to the following inclusion criteria:

a) has a focus on mental workload
b) has a focus on or uses EEG data
c) has a direct focus on reproducibility or evaluates a method across different settings, for example, in different tasks or at different times.

### 4.3 Analysis of the studies

Search in the databases produced 95 publications in total. The Scopus search produced 45 articles. Web of Science and ACM DL search yielded seven and one indifferent results, respectively. PubMed search produced 42 publications, six of which were distinct from the ones already found. As a result, we had 51 unique articles from these four databases. Only 13 of these 51 were considered relevant according to the eligibility criteria. This is an indication that few studies focus on reproducibility in the domain of mental workload classification from EEG signals.

Among the relevant thirteen studies, reproducibility was demonstrated for different electrode configurations and preprocessing pipelines (Mastropietro et al., 2023), for different settings of 2D and 3D environments (Kakkos et al., 2019), for a larger number of participants (Radüntz et al., 2020), for different tasks (Parekh et al., 2018; Boring et al., 2020; Sciaraffa et al., 2022), and over time (Gevins et al., 1998; Putze et al., 2013; Aricò et al., 2015, 2016b; Ortiz et al., 2020; Fox et al., 2022; Roy et al., 2022). Gevins et al. (1998) also tested their findings on separate tasks to check cross-task performance and finally on data from a new participant to observe cross-subject performance.

## 5 Establishing reproducible EEG machine learning pipelines: guidelines and checklist compilation

The objective of this section is to establish guidelines for constructing reproducible EEG machine learning pipelines. We compiled and tailored established guidelines from the reproducibility literature in both the EEG and machine learning research domains, supplemented these by our own contributions and structured the guidelines following CRISP-DM phases. Below, we discuss the guidelines per CRISP-DM phase. Table 1 outlines the finally resulting, complete list of checklist items and related research steps. Items on the checklist marked with a “†” rather reflect best practices or are related to generalizability, while those that are not marked directly affect reproducibility.

**Table 1:** Compiled checklist in relation to CRISP-DM phases: items on the checklist marked with a ^†^ reflect best practices or are related to generalizability while those that are not marked directly affect reproducibility.

**CRISP-DM phase**	**Research step**	**Compiled checklist**
Business Understanding	Problem definition	Problem/Scope statement
		Related literature^†^
Data Understanding	General	Dataset (name if public or private)
	Participant selection	Number of participants
		Participant recruitment method^†^ (e.g., direct mailing, advertisements)
		Participant sampling strategy (that constrain inclusion to a particular group/including population from which the participants were sampled)
		Age of participants
		Gender of participants
		Education level of participants
		Medications taken by the participants
		Prior/Current illness of participants
		Information on sleep deprivation
		Handedness of participants
		Consent of participants^†^
	Experimental setup	Type of EEG sensor/device (including make and model)
		Number of sensors
		Sensor locations
		Sampling rate
		Online filters (Type of filter and parameters)
		Electrode impedance
		Amplifier characteristics
		Measurement procedures
		Recording environment
		Participant seated or lying down status
	Experimental task information	Task description
		Characteristics of stimuli
		Instructions for the task
		Number of runs and sessions
		Clear timeline including - Timing of all stimuli/events - Intertrial intervals
		Software and hardware used for stimulus presentation
	Task-free recordings	Definition
		Timing
		Eyes open vs closed status
		If eyes open, fixation point usage
	Behavioral measures	Nature of the response
		Acquisition device and parameters
		Interface with EEG data and calibration procedures
		Errors and outliers handling
	Subjective measures	Subjective assessments recorded - Timing - Method
	Labeling	Definition
	Analysis	Recording length
		Statistical analysis to justify the number of trials and number of participants^†^
		Statistical analysis for descriptives of the collected measurements^†^
	Open sourcing	Open-sourced raw data with version control
		Open-sourced code for data collection with version control
		Open-sourced code/software for task execution with version control
Data Preparation	General	Flow of algorithms for preprocessing and transforming data
		Seeds for random number generators
	Sensor/Segment removal	For sensor/segment removal - Detection method and criteria - Interpolation parameters - Removed sensors/segments
	Artifact removal	For artifact removal/correction - Method - Range of parameters - Types of artifacts identified - Criteria to identify - Number/proportion of removed artifacts - Position of removed artifacts
		For signal–noise separation methods - Method - Parameters - Number of ICs - How non-brain ICs were identified - How back-projection was performed
	Downsampling	For downsampling - Method - Parameters
	Detrending	For detrending - Method - Parameters
	Filtering	For filtering - Filter type - Parameters
	Segmentation	For segmentation - Method - Parameters
	Baseline correction	For baseline correction - Method - Parameters
	Re-referencing	For re-referencing - Method
	Dimensionality Reduction	For dimensionality reduction - Method - Parameters
	Feature generation	For feature generation - Definition of features - Number of features - Method - Parameters
		Descriptive / Inferential statistics^†^ - The statistical method - Parameters
	Feature selection	Feature selection - Method - Number of selected features - Parameters - Selected features
	Data split	For data split (as training, validation and test) - Method - Parameters
		Separate test set^†^
	Feature transformation	Feature transformation (Normalization, Standardization,…) - Method - Parameters
		Feature transformations applied using training data?^†^
	Data augmentation	Data augmentation - Method - Parameters
		Data augmentation applied using training data?^†^
	Environment	Computing infrastructure
		Dependencies
	Open sourcing	Open-sourced preprocessed data with version control
		Open-sourced code for data preparation with version control
Modeling	General	ML problem (n-class MWL classification)
		Seeds for random number generators to prevent randomness in results
	Model	Algorithm name
		Explanation in detail, motivation and intended behavior^†^
		Loss function and parameters
		Regularization method and parameters
		Model structure
	Training strategy	Hyperparameters of the model
		Method for hyperparameter tuning
		Hyperparameter ranges for tuning
		Number of trials for hyperparameter tuning
		Selected hyperparameters
	Model training	Optimization method and parameters
		Number of training epochs/iterations
		Additional methods used during training and their parameters if any (e.g. early stopping, ..)
	Metrics	Metrics for model evaluation
		Chance-level values of metrics^†^
		Data split that the metrics are calculated on
		When the dataset is imbalanced, metrics other than accuracy^†^
		Confusion matrix^†^
	Environment	Computing infrastructure
		Dependencies
	Open sourcing	Open-sourced trained models with version control
		Open-sourced code for model training with version control
Evaluation	Statistical analysis	Statistical analysis for significance of results^†^ - Method - Parameters
	Metrics	Performance on independent test set^†^
		Computational resources for training^†^ (e.g. Model size, Training time, Power consumption, Carbon emissions)
	Conclusion	Relation of results to the problem statement
	Open sourcing	Open-sourced code for evaluation with version control
Deployment	Deployment Technique	Deployment techniques for limitedresources (e.g. quantization of the model, …) - Method - Parameters
	Metrics	Computational resources for inference^†^ (e.g. Model size, Inference time, Power consumption, Carbon emissions)
	Environment	Deployment infrastructure
		Dependencies
		Interfaces
		Schematic or sample view
	Deployment Test	Performance check^†^ (to see both development and production environments yield sufficiently similar results given identical input data)
		Performance monitoring method after deployment^†^

Researchers intending to use these guidelines should adapt them according to their specific methods if they are not covered. Moreover, applied methods should adhere to best practices and guidelines outlined in the relevant literature. Considering how an independent researcher can replicate the analysis or develop the same models by using only the content provided in the publications and supplementary materials is important. This requires a systematic approach during both the research process and the publication phase. As the field evolves and new methods emerge, any new essential information should be incorporated to align with the aforementioned focus on reproducibility. To achieve a comprehensive understanding necessary for replicating the results, a thorough comprehension of the methodologies employed is needed rather than relying solely on the direct execution of open-source code.

As a general approach for all phases, sharing of scripts and properties of the computation environment is required for complete reproducibility (Eglen et al., 2017). Text in code is expected to be human-readable, with necessary explanations provided in the comments. Reproducible code practices such as PEP-8 (Van Rossum et al., 2001) are suggested to use. Additionally, open-sourcing raw or at least preprocessed data with the definition of data and data structure should be ensured for full reproducibility. Shared resources ought to be easily accessible, and permanent access should be preferred.

### 5.1 Business understanding

In the context of machine learning research utilizing EEG data, stating the problem, including specific research questions or hypotheses and corresponding predictions (Keil et al., 2014), along with the related assumptions and literature provides a clear foundation and facilitates choosing the proper methodologies. A full grasp of the research problem, as well as the associated terminologies, is required to accomplish this phase. For this aspect, the following items are included in the checklist: “Problem/Scope statement” and “Related literature”.

### 5.2 Data understanding

This phase encompasses data collection, data description, data exploration, and data quality verification. Data collection and experiment design constitute a huge component of machine learning research with EEG data. Therefore, to better capture this important and multi-faceted process, we divide the “Data Collection” task of this phase into multiple research steps, namely, “General”, “Participant Selection”, “Experimental Setup”, “Experimental Task Information”, “Task-free Recordings”, “Behavioral Measures”, “Subjective Measures” and “Labeling”. Tables 2, 3 show the items of the checklist grouped by data collection research step together with their main reference. We mostly used items from Keil et al. (2014); Pernet et al. (2020) and Putze et al. (2022).

**Table 2:** Checklist items related to data collection-1.

**Research step**	**Compiled checklist**	**References**
General	Dataset (name if public or private)	
Participant selection	Number of participants	
	Participant recruitment method (e.g., direct mailing, advertisements)	Pernet et al., 2020; Putze et al., 2022
	Participant sampling strategy (that constrain inclusion to a particular group/including population from which the participants were sampled)	Pernet et al., 2020; Putze et al., 2022
	Age of participants	Keil et al., 2014; Pernet et al., 2020; Putze et al., 2022
	Gender of participants	Keil et al., 2014; Pernet et al., 2020; Putze et al., 2022
	Education level of participants	Keil et al., 2014
	Medications taken by the participants	Pernet et al., 2020
	Prior/Current illness of participants	
	Information on sleep deprivation	Kane et al., 2017
	Handedness of participants	
	Consent of participants	Pernet et al., 2020
Experimental setup	Type of EEG sensor/device (including make and model)	Keil et al., 2014; Putze et al., 2022
	Number of sensors	Keil et al., 2014
	Sensor locations	Keil et al., 2014; Putze et al., 2022
	Sampling rate	Keil et al., 2014; Putze et al., 2022
	Online filters (Type of filter and parameters)	Keil et al., 2014
	Electrode impedance	Keil et al., 2014; Putze et al., 2022
	Amplifier characteristics	Keil et al., 2014
	Measurement procedures	Keil et al., 2014
	Recording environment	Pernet et al., 2020; Putze et al., 2022
	Participant seated or lying down status	Pernet et al., 2020
Experimental task	Task description	
information	Characteristics of stimuli	Keil et al., 2014; Pernet et al., 2020
	Instructions for the task	Pernet et al., 2020; Putze et al., 2022
	Number of runs and sessions	Pernet et al., 2020
	Clear timeline including -Timing of all stimuli/events -Intertrial intervals	Keil et al., 2014; Putze et al., 2022
	Software and hardware used for stimulus presentation	Pernet et al., 2020; Putze et al., 2022

**Table 3:** Checklist items related to data collection-2.

**Research step**	**Compiled checklist**	**References**
Task-free recordings	Definition	
	Timing	
	Eyes open vs closed status	Pernet et al., 2020
	If eyes open, fixation point usage	Pernet et al., 2020
Behavioral measures	Nature of the response	Pernet et al., 2020
	Acquisition device and parameters	Pernet et al., 2020
	Interface with EEG data and calibration procedures	Pernet et al., 2020; Putze et al., 2022
	Errors and outliers handling	Pernet et al., 2020
Subjective measures	Subjective assessments recorded -timing -method	
Labeling	Definition	Putze et al., 2022

For recordings during real-life applications, such as driving or flying an airplane, marking recordings with respect to events would be more appropriate than using stimuli. In these cases, intertrial intervals or stimulus properties would not be applicable, and the checklist needs to be tailored to reflect such nuances.

EEG data is heavily dependent on the experimental settings and also the user's state of mind. Collecting additional data, such as subjective assessments and behavioral data, would be beneficial to mitigate the effects of these dependencies. These additional data can be instrumental during the evaluation of results, can be directly integrated into the models to normalize the data, or serve as separate input.

Labels should be clearly defined—e.g., whether workload labels are derived from task difficulty, subjective measures, or judgments by subject matter experts.

We consider data description, data exploration, and data quality verification tasks of CRISP-DM “Data Understanding” phase under the “Analysis” research step. For this step, we include in the checklist “Recording length”, “Statistical analyses to justify the number of trials and the number of participants” (Pernet et al., 2020), and “Statistical Analysis for descriptives of the collected measurements” (Keil et al., 2014). Exploratory data analysis to check for quality and descriptive analysis to better understand the data is advised to make informed decisions in the upcoming phases.

Furthermore, we also encourage the sharing of data to facilitate reproduction studies, along with the disclosure of source code/software used for data collection and task execution (Putze et al., 2022) under “Open-sourcing” step. An experiment cannot be reproduced to gather new data if the details of execution and data collection are left out. To prevent having to report every detail, standardized data collection methodologies and experiment software are required. This transparency enables independent labs to conduct the same experiment and replicate the results using their own data. Storing data in a standardized structure, such as EEG-BIDS (Pernet et al., 2019), is essential. Sharing of physiological data raises ethical considerations and informed consent of participants for the study, and usage or sharing of their data is obligatory during data collection (Hendriks et al., 2019).

### 5.3 Data preparation

This phase entails the tasks of selecting, cleaning, constructing, integrating, and formatting data in accordance with CRISP-DM. These tasks correspond to a large portion of the overall research process, from data preprocessing and feature generation to feature selection and feature transformation. The flow of steps used for preprocessing, feature generation, selection, and transformation should be well-defined (Keil et al., 2014) as well as the methods used and their related parameters. Seeds for random number generators need to be used and reported to prevent randomness in results (Azad et al., 2021).

We put special emphasis on data preprocessing steps, keeping in mind that there is not a common single pipeline and applications vary as well as the implementations and tools (Delorme et al., 2011; Bigdely-Shamlo et al., 2015; Robbins et al., 2020; Pernet et al., 2021; Delorme, 2023). Therefore, we aim to list the most commonly used techniques in our checklist (Table 1), in no particular order, leaving it to interested parties to tailor the same detailed approach for their own research. We took into account (Keil et al., 2014; Pernet et al., 2020; Putze et al., 2022) to list the most used preprocessing methods. More than one preprocessing pipeline can be used, yet consistency in feature generation, selection, and transformation steps is important throughout the study. All algorithms and corresponding parameters should be explicitly defined, and best practices for the applied methods should be followed. For example, de Cheveignè and Nelken (2019) reviewed filtering and explained how to choose the right filter. Keeping track of input and output data, data types, and data size at each research step supports coherence throughout the project.

Feature generation and feature selection are the next steps after preprocessing to prepare data for machine learning (Putze et al., 2022). Features are expected to be defined together with the method and parameters used to construct and select them. The total number of features, as well as the selected features and their number, should be stated. If descriptive or inferential statistics were analyzed, their method and parameters need to be reported (Keil et al., 2014).

Cross-validation is a widely employed technique to enhance model performance and generalizability. While cross-validation is typically performed during the modeling or evaluation phases, the initial step of splitting the data and setting aside a test set to prevent data leakage falls under the data preparation phase. In cases involving models that necessitate hyperparameter tuning, such as deep learning models or other parametric models, the hyperparameters are fine-tuned based on the performance metrics of validation sets. Consequently, an independent, unseen dataset for reporting the model's performance is required since the utilization of the validation set for hyperparameter optimization inherently introduces bias to the outcomes from the validation set. For these cases, it is common practice to divide the dataset into train, validation, and test sets. To ensure an unbiased estimate of the model's performance, the unseen test data should be set aside, excluding it from both model development and assessment until the final reporting stage to demonstrate the model's generalizability and avoid wrongly optimistic performance. Data split needs to return independent sets according to the task at hand to prevent leakage. For example, cross-subject estimation requires a subject-wise split, while cross-session models necessitate a session-specific split. Finding that models' performance cannot be reproduced from one individual to another, or from one session to another, will lead the research community to use other features or develop other types of models that can be generalized or to the conclusion that individual models are required. After the data is split into train, validation, and test sets, any data augmentation, transformation, or normalization should be executed using only the training set parameters to avoid potential data leakage. Brouwer et al. (2015) emphasizes the importance of selecting such parameters separately from the test set and using independent training and test sets as good classification practice. This approach establishes an unbiased common ground for the comparison of different algorithms. As a result, we add “Data split (Method, Parameters)” (Pineau et al., 2021), “Separate test set”, “Feature transformation (Method, Parameters)”, “Feature transformations applied using training data?”, “Data Augmentation (Method, Parameters)”, and “Data Augmentation applied using training data?” items to our checklist.

Overall, the process to obtain the feature sets should be provided in detail to prevent any gaps when generating them from scratch. Additionally, open-sourcing the scripts or providing the processed data would also mitigate these concerns (Gundersen and Kjensmo, 2018; Pineau et al., 2021; Putze et al., 2022).

### 5.4 Modeling

This phase consists of selecting the model, generating the test design, and building and assessing the model. Best practices in the literature need to be followed for these tasks. For example, Bengio (2012) provides practical recommendations for training deep neural networks.

When selecting a model, one should provide a detailed explanation of the rationale behind the choice and its intended behavior (Gundersen and Kjensmo, 2018). In the case of opting for an existing validated method, the report should reference relevant packages, functions, or repositories. Additionally, one should explicitly state model parameters, including the loss function, regularization, other internal settings, and model structure, if applicable. Similar to data preparation, the use and reporting of random number generator seeds for reproducibility and obtaining deterministic results should also be ensured.

Generating the test design is inherent to the training strategy. For parametric methods, hyperparameters of the model and the method for tuning them together with their ranges and number of trials should be reported, including the selected hyperparameters (Pineau et al., 2021; Putze et al., 2022). After the test design, the upcoming step in the project is model training. The optimization method and its parameters as well as the number of training epochs or iterations (Pineau et al., 2021) should be defined at this stage. Additional techniques utilized during training, such as early stopping, need to be stated with the relevant parameters (Bengio, 2012).

Once test design and model building are completed, generated models need to be assessed technically and compared to choose the best model or models. Evaluation metrics should be defined with their reasoning (Pineau et al., 2021; Putze et al., 2022) and their chance-level values need to be included. Naive baseline models and naive predictions are important to build, in particular when dealing with class imbalanced datasets. A naive model, which generates the majority class label at all times could imply whether the developed model is useful. Chance-level values can be extracted by using random estimators.

Additionally, attention is required when the model performance is to be compared with baseline parametric models proposed in the literature. Using model parameters as they are would lead to wrong conclusions since they would be tuned specifically to the dataset of the original study. Parameters of baseline models should also be adjusted, if possible, with the methodology provided in the original paper to perform a fair comparison (Sculley et al., 2018). This would be possible if the reference study is also reproducible.

When reporting the results, the data split that the metrics are calculated on (train, validation, or test) must be explicitly stated. When the dataset is imbalanced, metrics other than accuracy should be used. Using confusion matrices is encouraged to identify regions where the model does not fit completely. This is specifically relevant for machine learning with EEG since these datasets are usually small. Using a combination of complementary metrics rather than relying on a single metric helps a more extensive understanding of machine learning performance (Canbek et al., 2021). Moreover, categories of data can be used to break down performance measures to understand the results in different regions (Sculley et al., 2018).

Machine learning models are prone to computational environment changes; therefore, a description of the computing hardware and software infrastructure needs to be presented together with the dependencies, including external libraries and their versions or virtual environment with all dependencies (Gundersen and Kjensmo, 2018; Pineau et al., 2021). It would be beneficial to test whether the same set of packages works on other related environments, such as on a different device or operating system, before moving on to deployment.

In conclusion, similar to the approach in the Data Preparation phase, the process for modeling should be described in detail so that an independent researcher can reproduce the results. Open-sourcing the modeling scripts and providing the trained models are also encouraged for details that may have been left out or to mitigate misunderstandings from written text.

Data Preparation and Modeling phases are managed iteratively since the two phases affect each other closely.

### 5.5 Evaluation

In this phase, results are discussed in line with the research questions or hypotheses stated in the Business Understanding phase.

Due to high efforts required in EEG data collection, the number and variety of participants are usually low. Therefore, the distribution of the whole population cannot be normally captured equally within data splits. Model selection, assessment, and comparison need to be performed on validation sets since training sets are used for model training. After finding the best model, to generate an unbiased estimate of the performance, an independent test set should be used for reporting. This test set should not be included in model development or selection. Results on the test set need to be presented to check for generalizability and prevent misleading optimistic findings. For the most reliable results, nested cross-validation is recommended (Pernet et al., 2020). Finally, statistical analysis should be performed to ascertain the significance of results. For comparison of classifiers, appropriate statistical tests need to be used (Müller-Putz et al., 2008), such as (non-parametric) Wilcoxon signed ranks and the Friedman test (Demšar, 2006).

Recently, Strubell et al. (2020) emphasized the increase in computational resources of machine learning research as larger models are trained with larger amounts of data for performance improvement. They advise researchers to report training time and sensitivity to hyperparameters. Moreover, they are expected to prioritize computationally efficient hardware and algorithms and be mindful of energy sources powering their computing. Schwartz et al. (2020) proposed Green AI, where the focus of research would be efficiency rather than accuracy. This approach aims to reduce the environmental impact of model training and the entry barriers to the field, both caused by increased computational resource requirements. Computational resources for training, such as model size, run time or power consumption, and carbon emissions, need to be reported to promote responsible AI that is energy-efficient. Releasing code and data or models also helps reduce carbon emissions as it will reduce the energy spent on replicating the results by other researchers (Henderson et al., 2020). Open-sourcing the scripts for evaluation is also encouraged to perform appropriate comparisons.

After the evaluation of results in this phase, the process is reviewed, and the next steps are determined as to reiterate from the Business Understanding phase or move on with Deployment.

### 5.6 Deployment

While deploying models is essential for real-world applications, current EEG (workload) research tends to focus more on developing new methods for classification or data processing approaches rather than on deployment specifics. When models are deployed, it is important to provide details about the deployment hardware, software infrastructure, and dependencies. Additionally, reporting required computational resources for inference, such as inference time, power consumption, and carbon emissions, is necessary. Challenges arise with the growing sizes of recent models, like those in natural language or image processing, as they may pose difficulties in deployment due to constraints on size or cost in practical applications. Deployment techniques, including low-rank factorization or model quantization, along with computational optimization methods, can be employed to address these challenges (Huyen, 2022). If such techniques are used, it is important to report the methods and parameters involved.

Interfaces and schematic or sample views need to be presented for a good understanding of the application. The performance of the model is required to be verified to yield sufficiently similar results in both development and production environments given identical input.

In an optimal scenario, for results to be deemed appropriate for a real-world application, developed models should exhibit consistent and acceptable performance across diverse subjects and various time frames. If the conditions permit, it would be best to model and evaluate these aspects to demonstrate the generalizability before deployment. Between the development and deployment environments, data flow must be consistent end-to-end, from preprocessing the data to generating the features and inferring the results. Moreover, after deployment, performance needs to be monitored and maintained continuously to prevent any problems and model drift. The method to achieve this monitoring can be reported for transparency.

## 6 Reproducibility in machine learning models to predict mental workload using EEG

We performed a comprehensive literature review to assess the extent to which the aspects in the checklist (Table 1) have been implemented within the domain of mental workload classification studies utilizing EEG data. Although this section is dedicated to assessing the reproducibility status of mental workload classification using EEG, the guidelines and checklist have the potential to be applicable to most other EEG machine learning studies.

### 6.1 Literature search strategy

**Figure 2:**
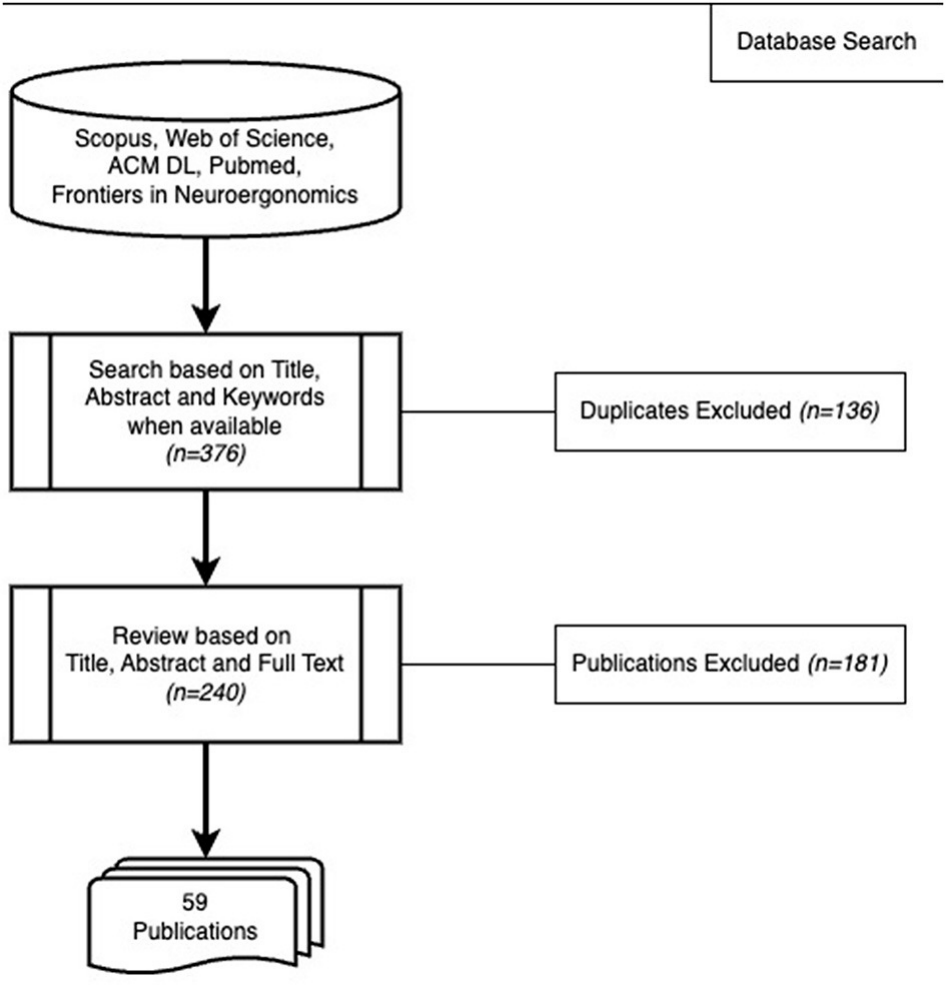
Search strategy for literature review 2.

Figure 2 shows our search strategy. During phase I, we searched in titles, abstracts, and keywords in Scopus, Web of Science, ACM Digital Library (ACM DL), and Pubmed databases with the following search term: “*Machine Learning” AND “EEG” AND (“Workload” OR “Cognitive Load” OR “Mental Effort” OR “Mental Load”)* in September 2023. We did not include limits on the language at this stage. We also searched in the “Frontiers in Neuroergonomics” journal from the webpage as at the time of the search, this journal was not yet indexed in the aforementioned databases and its scope directly entails our topic. We searched in full-text for this journal because a search based on only titles, abstracts, and keywords was not possible.

### 6.2 Eligibility criteria

In phase II, we selected publications according to the criteria given in Table 4. All inclusion criteria are domain-specific, and all exclusion criteria are generic.

**Table 4:** Inclusion and exclusion criteria.

**Inclusion criteria**		**Exclusion criteria**	
I1	Models mental workload/cognitive load	E1	Original language is not English
I2	Applies machine learning	E2	Full text is not available
I3	Uses only EEG data features	E3	Review paper
I4	Uses at least one classifier		
I5	Uses EEG data at the sensor level		

### 6.3 Analysis of the studies

The search in the databases produced 376 publications in total. The Scopus search produced 210 articles. Web of Science yielded 73 papers, but none of them were different. Only one out of ten results from ACM DL was distinct. Pubmed search produced 61 results, 7 of which were new. The search in Frontiers in Neuroergonomics generated 22 results. As a result, we had 240 unique articles from these four databases when duplicates were removed. From these, 59 publications met the eligibility criteria (Table 5). In phase III, we inspected these publications in detail. We showcase the status of reproducibility among the selected papers by following the checklist given in Table 1. Results of phase III are presented in Section 6.4.

**Table 5:** List of publications.

**References**	**Year**	**Title**	**Journal/Conference name**
Taheri Gorji et al. (2023)	2023	Using machine learning methods and EEG to discriminate aircraft pilot cognitive workload during flight	Scientific Reports
Chiang et al. (2023)	2023	Using EEG signals to assess workload during memory retrieval in a real-world scenario	Journal of Neural Engineering
Zhu et al. (2023)	2023	Recognition of Pilot Mental workload in the Simulation Operation of Carrier-based Aircraft Using the Portable EEG	ACM International Conference Proceeding Series
Zemla et al. (2023)	2023	Modeling of Brain Cortical Activity during Relaxation and Mental Workload Tasks Based on EEG Signal Collection	Applied Sciences (Switzerland)
Zhang et al. (2023)	2023	A Mental Workload Classification Method Based on GCN Modified by Squeeze-and-Excitation Residual	Mathematics
Guan et al. (2023)	2023	Cross-Task Mental Workload Recognition Based on EEG Tensor Representation and Transfer Learning	IEEE Transactions on Neural Systems and Rehabilitation Engineering
Teymourlouei et al. (2023)	2023	Decoding EEG Signals with Visibility Graphs to Predict Varying Levels of Mental Workload	2023 57th Annual Conference on Information Sciences and Systems, CISS 2023
Kingphai and Moshfeghi (2023)	2023	On Time Series Cross-Validation for Deep Learning Classification Model of Mental Workload Levels Based on EEG Signals	Lecture Notes in Computer Science (including subseries Lecture Notes in Artificial Intelligence and Lecture Notes in Bioinformatics)
Zheng et al. (2023)	2023	Inter-subject cognitive workload estimation based on a cascade ensemble of multilayer autoencoders	Expert Systems with Applications
Yedukondalu and Sharma (2023)	2023	Cognitive load detection using circulant singular spectrum analysis and Binary Harris Hawks Optimization based feature selection	Biomedical Signal Processing and Control
Patel et al. (2022)	2022	Optimal classification of N-back task EEG data by performing effective feature reduction	Sadhana - Academy Proceedings in Engineering Sciences
Albuquerque et al. (2022)	2022	Estimating distribution shifts for predicting cross-subject generalization in electroencephalography-based mental workload assessment	Frontiers in Artificial Intelligence
Sciaraffa et al. (2022)	2022	Evaluation of a New Lightweight EEG Technology for Translational Applications of Passive Brain-Computer Interfaces	Frontiers in Human Neuroscience
Wu et al. (2022)	2022	Self-Paced Dynamic Infinite Mixture Model for Fatigue Evaluation of Pilots' Brains	IEEE Transactions on Cybernetics
Raufi and Longo (2022)	2022	An Evaluation of the EEG Alpha-to-Theta and Theta-to-Alpha Band Ratios as Indexes of Mental Workload	Frontiers in Neuroinformatics
Zhao et al. (2022)	2022	Assessing Distinct Cognitive Workload Levels Associated with Unambiguous and Ambiguous Pronoun Resolutions in Human–Machine Interactions	Brain Sciences
Yedukondalu and Sharma (2022)	2022	Cognitive load detection using Binary salp swarm algorithm for feature selection	2022 IEEE 6th Conference on Information and Communication Technology, CICT 2022
Liu et al. (2022)	2022	EEG based Mental Workload Assessment by Power Spectral Density Feature	2022 IEEE International Conference on Mechatronics and Automation, ICMA 2022
Babu et al. (2022)	2022	Analysis of Mental Task Ability in Students based on Electroencephalography Signals	SPICES 2022 - IEEE International Conference on Signal Processing, Informatics, Communication and Energy Systems
Zanetti et al. (2022)	2022	Real-Time EEG-Based Cognitive Workload Monitoring on Wearable Devices	IEEE Transactions on Biomedical Engineering
Hussain et al. (2021)	2021	Driving-induced neurological biomarkers in an advanced driver-assistance system	Sensors
Sharma et al. (2021)	2021	Cognitive performance detection using entropy-based features and lead-specific approach	Signal, Image and Video Processing
Kakkos et al. (2021)	2021	EEG Fingerprints of Task-Independent Mental Workload Discrimination	IEEE Journal of Biomedical and Health Informatics
Rahman et al. (2021)	2021	Prediction and Detection in Change of Cognitive Load for VIP's by A Machine Learning Approach	3rd IEEE International Conference on Artificial Intelligence in Engineering and Technology, IICAIET 2021
Kutafina et al. (2021)	2021	Tracking of mental workload with a mobile eeg sensor	Sensors
Shao et al. (2021)	2021	FINE-GRAINED and MULTI-SCALE MOTIF FEATURES for CROSS-SUBJECT MENTAL WORKLOAD ASSESSMENT USING BI-LSTM	Journal of Mechanics in Medicine and Biology
Balamurugan et al. (2021)	2021	Brain–computer interface for assessment of mental efforts in e-learning using the nonmarkovian queueing model	Computer Applications in Engineering Education
Ved and Yildirim (2021)	2021	Detecting Mental Workload in Virtual Reality Using EEG Spectral Data: A Deep Learning Approach	Proceedings - 2021 4th IEEE International Conference on Artificial Intelligence and Virtual Reality, AIVR 2021
Cheng et al. (2021)	2021	The Cognitive Load Evaluation Based on EEG with K-Nearest Neighbor Algorithm	ISPACS 2021 - International Symposium on Intelligent Signal Processing and Communication Systems: 5G Dream to Reality, Proceeding
Sciaraffa et al. (2021)	2021	Mental Effort Estimation by Passive BCI: A Cross-Subject Analysis	Proceedings of the Annual International Conference of the IEEE Engineering in Medicine and Biology Society, EMBS
Diwakar et al. (2020)	2020	Deep Learning Identifies Brain Cognitive Load Via EEG Signals	2020 IEEE 17th India Council International Conference, INDICON 2020
Do et al. (2020)	2020	Estimating the cognitive load in physical spatial navigation	2020 IEEE Symposium Series on Computational Intelligence, SSCI 2020
Pandey et al. (2020)	2020	Mental Workload Estimation Using EEG	Proceedings - 2020 5th International Conference on Research in Computational Intelligence and Communication Networks, ICRCICN 2020
Becerra-Sánchez et al. (2020)	2020	Feature selection model based on eeg signals for assessing the cognitive workload in drivers	Sensors
Qiao and Bi (2020)	2020	Ternary-task convolutional bidirectional neural turing machine for assessment of EEG-based cognitive workload	Biomedical Signal Processing and Control
Plechawska-Wójcik et al. (2019)	2019	A three-class classification of cognitiveworkload based on EEG spectral data	Applied Sciences (Switzerland)
Tao et al. (2019)	2019	Individual-specific classification of mental workload levels via an ensemble heterogeneous extreme learning machine for EEG modeling	Symmetry
Gu et al. (2019)	2019	EEG based mental workload assessment via a hybrid classifier of extreme learning machine and support vector machine	Chinese Control Conference, CCC
Yin et al. (2019)	2019	Physiological-signal-based mental workload estimation via transfer dynamical autoencoders in a deep learning framework	Neurocomputing
Zhang et al. (2019a)	2019	Spectral and Temporal Feature Learning with Two-Stream Neural Networks for Mental Workload Assessment	IEEE Transactions on Neural Systems and Rehabilitation Engineering
Di Flumeri et al. (2019)	2019	EEG-Based Workload Index as a Taxonomic Tool to Evaluate the Similarity of Different Robot-Assisted Surgery Systems	Communications in Computer and Information Science
Sciaraffa et al. (2019)	2019	On the Use of Machine Learning for EEG-Based Workload Assessment: Algorithms Comparison in a Realistic Task	Communications in Computer and Information Science
Zhang et al. (2019b)	2019	Learning Spatial-Spectral-Temporal EEG Features With Recurrent 3D Convolutional Neural Networks for Cross-Task Mental Workload Assessment	IEEE Transactions on Neural Systems and Rehabilitation Engineering
Parekh et al. (2018)	2018	Investigating the generalizability of EEG-based cognitive load estimation across visualizations	Proceedings of the 20th International Conference on Multimodal Interaction, ICMI 2018
Blanco et al. (2018)	2018	Quantifying cognitive workload in simulated flight using passive, dry EEG measurements	IEEE Transactions on Cognitive and Developmental Systems
Appriou et al. (2018)	2018	Towards robust neuroadaptive HCI: Exploring modern machine learning methods to estimate mental workload from EEG signals	Conference on Human Factors in Computing Systems - Proceedings
Jiao et al. (2018)	2018	Deep Convolutional Neural Networks for mental load classification based on EEG data	Pattern Recognition
Saha et al. (2018)	2018	Classification of EEG signals for cognitive load estimation using deep learning architectures	Lecture Notes in Computer Science (including subseries Lecture Notes in Artificial Intelligence and Lecture Notes in Bioinformatics)
Cheema et al. (2018)	2018	Mental workload estimation from EEG signals using machine learning algorithms	Lecture Notes in Computer Science (including subseries Lecture Notes in Artificial Intelligence and Lecture Notes in Bioinformatics)
Dai et al. (2017)	2017	Mental workload classification in n-back tasks based on single-trial EEG	Yi Qi Yi Biao Xue Bao/Chinese Journal of Scientific Instrument
Yin and Zhang (2017)	2017	Cross-session classification of mental workload levels using EEG and an adaptive deep learning model	Biomedical Signal Processing and Control
Zhou et al. (2017)	2017	Monitoring cognitive workload in online videos learning through an EEG-based brain-computer interface	Lecture Notes in Computer Science (including subseries Lecture Notes in Artificial Intelligence and Lecture Notes in Bioinformatics)
Abrantes et al. (2017)	2017	Classification of EEG features for prediction of working memory load	Advances in Intelligent Systems and Computing
Aricò et al. (2016a)	2016	Adaptive automation triggered by EEG-based mental workload index: A passive brain-computer interface application in realistic air traffic control environment	Frontiers in Human Neuroscience
Aricò et al. (2015)	2015	Reliability over time of EEG-based mental workload evaluation during Air Traffic Management (ATM) tasks	Proceedings of the Annual International Conference of the IEEE Engineering in Medicine and Biology Society, EMBS
Ke et al. (2015)	2015	Towards an effective cross-task mental workload recognition model using electroencephalography based on feature selection and support vector machine regression	International Journal of Psychophysiology
Dimitriadis et al. (2015)	2015	Cognitive Workload Assessment Based on the Tensorial Treatment of EEG Estimates of Cross-Frequency Phase Interactions	Annals of Biomedical Engineering
Penaranda and Baldwin (2012)	2012	Temporal factors of EEG and artificial neural network classifiers of Mental Workload	Proceedings of the Human Factors and Ergonomics Society
Grimes et al. (2008)	2008	Feasibility and pragmatics of classifying working memory load with an Electroencephalograph	Conference on Human Factors in Computing Systems - Proceedings

### 6.4 Reproducibility analysis

By inspecting the selected 59 publications based on the full text according to the guidelines presented in Table 1, we aimed to establish which elements of the guidelines in our list are commonly adhered to, and which elements of the guidelines in our list are commonly ignored in machine learning research that models mental workload using EEG.

**Figure 3:**
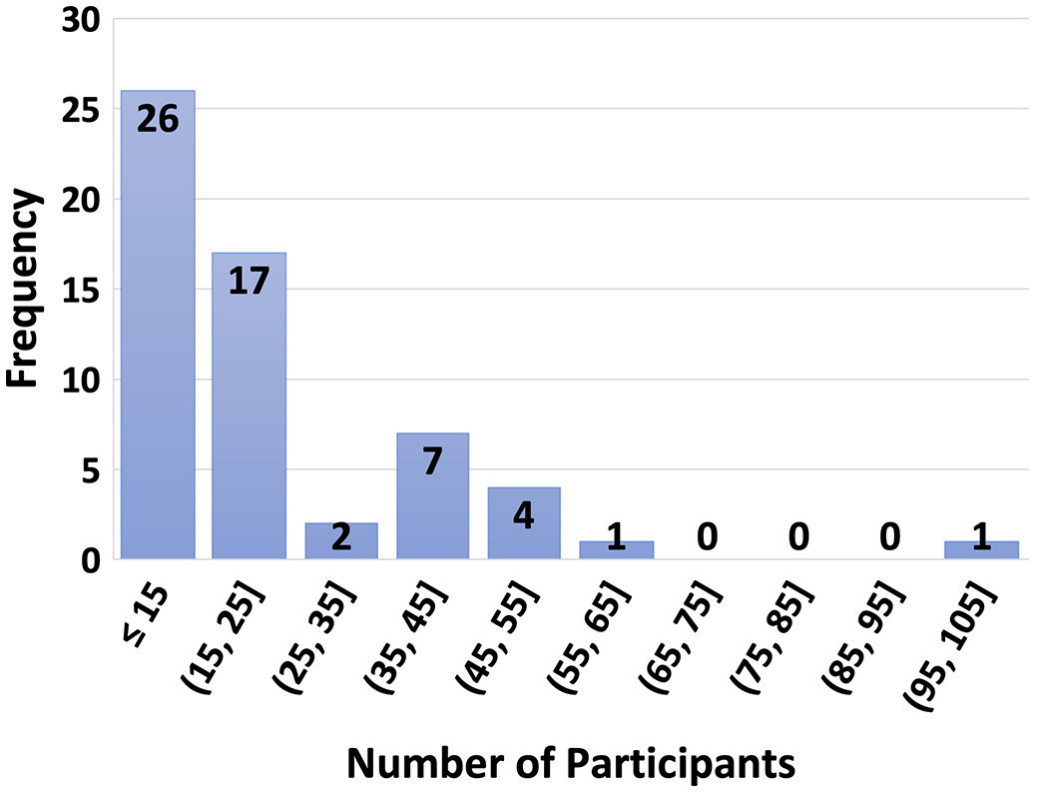
Number of participants.

1. Business Understanding: Related to the Business Understanding phase, we considered “Problem/Scope statement” present when the objective of the paper was stated in the Abstract or Introduction sections. Additionally, if the problem was described in the Introduction with references or a separate “Literature Review” section, “Related literature” was marked as present. According to our analysis, all publications defined the problem and presented relevant literature, although the extent of their coverage differed.
2. Data Understanding: Checklist items regarding Participant Selection, Experimental Setup, Experimental Task Information, Task-free recordings, Behavioral Measures, Subjective Measures, Labeling, and Analysis research steps are evaluated for the selected papers. Table 6 shows the reported percentages of the checklist items related to the participants, experiment, labeling, and statistical analysis. Table 7 shows the reported percentages of additionally collected data, namely, task-free recordings, behavioral and subjective measures.
  Sixteen of the publications used an open dataset. When a publication referenced an open dataset, we checked the relevant publication to analyze if the checklist items were reported. Additionally, when more than one dataset was used, we marked an item present if it was included for at least one of the datasets. We considered the “Education level of participants” provided if the “Participant sampling strategy” stated information about education level, for example, graduate students or pilots. “Prior/Current illness of participants” was marked as reported if it was explicitly stated or the participants were stated to be healthy. Participants identified as healthy were presumed to be free from medication use.
  “Amplifier characteristics” were considered present when an amplifier model or amplifier properties such as channel number or time constant were specified. “Participant seated or lying down status” was marked as present if it was explicitly stated or it could be inferred from the recording environment or the task.
  “Recording length” was considered given if it was explicitly stated or it could be calculated from given information.
  Characteristics of stimuli were marked as given when it was explicitly stated or it could be inferred from the task description, for example, visual or auditory stimuli. Detailed instructions for the experimental task are required for reproduction. We marked a study to have reported instructions, whether the instructions were related to the experiment execution or physical restraints, such as refraining from movement. Even if most of the studies (80%) reported instructions, capturing all information in reports to enable the execution of tasks by other researchers is hard. Although 73% had their own recording and dataset, only three of them had the raw data available upon request, and two of them had the preprocessed data available. Open datasets and standardized data collection methodologies and experiment settings should be established to overcome most of these challenges. Similarly, open-source codes help to reproduce the methodologies and provide a common baseline for comparisons, yet only two of the publications shared their data processing repositories. In addition to open-sourcing, authors need to be willing to help other researchers perform experiments. Expanding the knowledge base toward generalizable models for real-life applications is possible by achieving a collaborative research environment.
3. Data preparation: In our analysis, we considered the “flow of algorithms” included even if it was listed in one sentence. Flow of algorithms used to preprocess data, to generate and select features and to develop models were reported for most of the studies (93%). 'Seeds for random number generators' were marked as given when they were stated explicitly or the code was open. Two publications, which shared their codes, were consequently marked as reporting them and one publication provided the seed number.
  Table 8 shows the status of preprocessing items. The percentage of application of the research steps and the percentage that parameters were reported among them are presented. Similar to performing the experiments, data preparation, and modeling would be best understood by independent researchers when code and data are shared to prevent having to state all parameters in detail.
  Feature generation was unclear for three of the publications, and the number of features was not explicitly stated for 29% of the publications. Feature generation method and parameters were not explicitly stated for 32% and 41% of the publications, respectively. Thirty four% of the publications performed descriptive statistics and the method was specified for 85% of them.
  53% of the publications performed feature selection, and 60% of those that performed feature selection indicated the number of selected features. 93% of the publications stated method for data splits, and 25% among them listed their parameters in the form of percentages, fold numbers, or session-based splits.
  Fifteen% and 42% of the publications provided information about the computing infrastructure and dependencies, respectively. “Dependencies” were marked given even if only one software package or software was stated (e.g., Python, scikit-learn, Tensorflow, EEGLAB (version 14.2.0), MATLAB2019b).
4. Modeling: For the modeling phase, 64% of the studies explain the algorithm used and the motivation to apply it. Sixty-nine% of the publications state the hyperparameters, 56%, 44% and 41% of them report the method for hyperparameter tuning, state ranges of the hyperparameters, and present selected hyperparameters, respectively. Only one of the publications that use methods other than grid search reported the number of trials for hyperparameter tuning. Here, we exclude grid search as the number of trials for it can be deduced from parameter ranges. Detailed information on models or model training, such as loss function, regularization, model structure, optimizer, or number of training epochs/iterations, are not applicable to all models. Therefore, they could only be investigated where applicable. To present the general situation, we extracted their reporting percentages without considering the related models. Optimization method, number of training epochs/iterations, and additional methods used during training were reported for 22%, 24%, and 17 % of the publications, respectively.
  All publications except one report the metrics used, and five of them state the chance-level value. Data split that the metrics are calculated on is not clearly explained for 17% of the publications. Additionally, 20% report confusion matrices.
  When we consider computational environment and open sourcing, 29% and 44% report computing infrastructure and dependencies, respectively. None of them open-source their trained models and only three of them open-source their code for modeling.
5. Evaluation: Statistical analysis for significance of results was carried out by 42% of the publications, 68% of which also included parameters such as alpha parameter, confidence interval, or p-value. During the Evaluation phase, an unseen test set to report the performance of the model is mandatory for unbiased estimates and to present the generalizability of results. However, 39% of the studies hold out a test set and only 34% report the results on the test set. For EEG modality, setting aside an unseen test set can be difficult considering the limited amount of data and low number of participants. EEG data collection is time-consuming, and it may be difficult to find participants who satisfy the inclusion criteria and are willing to participate in the experiment. To illustrate this, Figure 3 shows a histogram of the number of participants where most studies include 15 or less participants. One publication did not state the number of subjects.
  Few studies (20%) report the computational resources for training such as model size, training or inference times, power consumption, and carbon emissions. We consider these resources reported even when one of these types of data is presented. These aspects are closely related to both the limitations of the deployment environment and sustainable AI.
  All publications related their results to the problem statement. Three of them open-sourced their code for evaluation.
6. Deployment: Only one of the publications considered deployment. Deployment techniques, computational resources required for inference, deployment environment, and deployment tests need to be considered after finalizing the model in the development environment. Deployed systems must retain their performance and be reliable, scalable, maintainable, and adaptable (Huyen, 2022).

**Table 6:** Reported percentages of checklist items in the data understanding phase-1.

**Research step**	**Checklist item**	**Percentage reported (%)**
Participant selection	Participant recruitment method	8
	Participant sampling strategy	75
	Age of participants	71
	Gender of participants	76
	Education level of participants	59
	Medications taken by the participants	34
	Prior/Current illness of participants	64
	Information on sleep deprivation	7
	Handedness of participants	42
	Consent of participants	66
Experimental setup	Type of EEG sensor/device	86
	Number of Sensors	98
	Sensor Locations	85
	Sampling rate	88
	Online filters	15
	Electrode impedance	34
	Amplifier characteristics	31
	Measurement procedures	37
	Recording environment	73
	Participant seated or lying down status	75
Experimental Task Information	Task Description	100
	Characteristics of stimuli	54
	Instructions for the task	81
	Number of runs and sessions	93
	Timing of all stimuli/events	68
	Intertrial intervals	61
	Software and hardware for stimulus presentation	56
Labeling	Definition	98
Analysis	Recording Length	86
	Statistical analysis to justify the number of trials and number of participants	2
	Statistical analysis for descriptives of the collected measurements	39

**Table 7:** Reported percentages of checklist items in the data understanding phase-2: the third column refers to percentages of the subset in the second column.

**Research step**	**Usage percentage (%)**	**Parameter percentages (%)**
Task-free recordings	54	Timing: 84 Eyes open or closed status: 69 If eyes open, fixation point usage: 13
Behavioral measures	51	Acquisition device: 43 Interface with EEG data and calibration procedures: 23 Method for errors and outlier handling: 3
Subjective measures	32	Timing: 100 Method: 100

**Table 8:** Reported percentages of checklist items in the data preparation phase: the third column refers to percentages of the subset in the second column.

**Research step**	**Usage percentage (%)**	**Parameter percentages (%)**
Sensor/segment removal	19	Interpolation: 18 Removed sensors: 55
Artifact removal/correction	39	Range of parameters: 13 Types of artifacts identified: 69 Criteria to identify: 30 Number/proportion of removed artifacts: 4 Position of removed artifacts: 0
Signal-noise separation methods	39	Parameters: 17 Number of ICs: 4 How non-brain ICs were identified: 30 How back-projection was performed: 17
Downsampling	17	Method: 40 Parameters: 80
Detrending	5	Method: 100 Parameters: 0
Filtering	76	Filter type: 100 Parameters: 96
Segmentation	80	Method: 100 Parameters: 96
Baseline correction	3	Method: 100 Parameters: 100
Re-referencing	19	Method: 100
Dimensionality Reduction	2	Method: 100 Parameters: 0
Feature Transformation	22	Method: 100 Parameters: 77 Applied using training data?: 31
Data Augmentation	12	Method: 100 Parameters: 43 Applied using training data?: 14

## 7 Discussion

This study introduced guidelines, compiled in a checklist aligned with the CRISP-DM framework, for improving the reproducibility of machine learning research utilizing EEG data. A systematic evaluation of EEG mental workload studies shed light on commonly employed strategies, frequently overlooked aspects, and the existing gaps that impede progress toward achieving reproducible science for practical applications.

The key revelation from our analysis is the prevalent limitation in reproducibility across the examined studies. Notably, a significant number of publications fall short in reporting performance on unseen test data, an important aspect that is informative of the model's generalizability. This omission poses a potential problem to the applicability of these models in diverse settings and under varying conditions.

Furthermore, our investigation reveals that only a minority of studies share essential resources, such as data or scripts, crucial for achieving full reproducibility. Given the inherent complexity of capturing every detail in the machine learning pipeline, the open sharing of data and code emerges as a key factor in increasing the credibility of models. This not only builds trust but also helps speed up progress by making it easier to understand new research, saving time on reproducing results, and creating starting points for future work.

A third noteworthy finding is the inadequate reporting of resources essential for training and inference processes. Now that the detrimental environmental effects of AI are becoming increasingly clear, reporting the training and inference times, power consumption, and carbon emissions has become a recommended practice. The inclusion of such information is important for fostering environmentally conscious practices in machine learning research. Deployment techniques to compress models or optimize inference are being developed. With only one study found in our survey specifically addressing deployment considerations, there is an apparent need to study and discuss deployment strategies for EEG classification using machine learning.

Our study has several implications.

Firstly, the introduction of a guideline and checklist, aligned with the CRISP-DM framework, provides a foundational framework for researchers in the field. Adhering to these guidelines will result in a clearer understanding and validation of the methodologies employed, enable the reduction of errors, and improve the credibility and reliability of machine learning studies utilizing EEG data, their authors, and the scientific field as a whole, promoting better scientific practices and accelerated progress.

Secondly, by using the introduced checklist, models can be more fairly compared, ensuring a comprehensive evaluation. With models being compared more fairly, the results and experiments become more transparent and interpretable.

Thirdly, key findings from the reproducibility assessment highlight areas for improvement and future work.

While the present study has contributed valuable insights, there are limitations and promising paths for future research.

Search terms for the systematic literature reviews could be added to enhance coverage and inclusivity. Terms could be expanded to include similar words, such as “Electroencephalography” in addition to “EEG” and “Classification” in addition to “Machine Learning”. Additionally, in the first literature review, not all studies examining reproducibility will have emerged using our terms "reproducibility", "replicability", and "generalizability". Moreover, we focus on the reproducibility status of mental workload estimation studies using EEG. This work could still be extended to include the reproducibility status of EEG studies in general.

Our study focused on mental workload estimation studies. The proposed checklist has the potential to be applied to EEG machine learning studies in general, in particular mental state monitoring in a broader sense. Future work could explore reproducibility of machine learning studies using EEG across various domains, e.g., mental states besides workload, therewith broadening the scope of the reproducibility results and checking in detail for applicability of the proposed checklist across domains. In addition, it would be of interest to examine how reproducibility of different aspects depends on the working domain or expertise of the authors. Mental workload estimation is an interdisciplinary topic. Authors' background and main expertise likely affect the degree of reproducibility of different aspects, and interdisciplinary teams will likely increase the overall quality of reproducibility.

Transparency and explainability are now integral components of Responsible AI, and are as such requested in various standards, recommendations, and regulations, including the EU AI Act, OECD AI principles, and ISO/IEC 42001:2023. These principles are also catalyzing the acceleration of reproducible studies in the field of machine learning. In the future, the proposed guidelines could incorporate Responsible AI aspects, such as the growing significance of explainability features in model development. These features are increasingly becoming essential, even mandated, in the regulations of certain countries. Further research is needed to explore and address deployment strategies, especially considering the environmental impact and practical applications.

The current study did not account for the time frame of the considered papers. A crucial aspect for future exploration involves investigating whether reproducibility and other good practices have undergone changes over time. Given the increasing topic-related standards and publication requirements in recent years, it is pertinent to examine if these shifts have influenced reproducibility in more recent papers.

In conclusion, the proposed guidelines for reproducible machine learning research using EEG, as well as the overview of the current state of the literature regarding reproducibility, have the potential to support and motivate the community to further improve the current state of affairs. Our findings highlight the necessity for a change in research methods, putting a focus on transparency, sharing data openly, and reporting resources in detail. Tackling these issues is crucial for moving the field forward, building trust in models, improving the quality of studies, and lessening the environmental impact of machine learning applications.

## Author contributions

GD: Writing – original draft. TT: Supervision, Writing – review & editing. A-MB: Supervision, Writing – review & editing.

## Footnotes

^1^ https://documentation.sas.com/doc/en/emref/14.3/n061bzurmej4j3n1jnj8bbjjm1a2.htm

^2^ https://learn.microsoft.com/en-us/azure/architecture/data-science-process/lifecycle
